# Using wavelet decomposition to determine the dimension of structures from projected images

**DOI:** 10.1073/pnas.2534122123

**Published:** 2026-03-23

**Authors:** Svitlana Mayboroda, David N. Spergel

**Affiliations:** ^a^Department of Mathematics, ETH Zurich, Zürich 8092, Switzerland; ^b^Flatiron Institute, New York, NY 10011

**Keywords:** fractals, interstellar medium, data analysis

## Abstract

Mesoscale structures in turbulent media can often be described as fractional dimensional across a wide range of scales. The goal of this paper is to determine the structure’s dimension from a projected image. Our method exploits the laws of scaling of wavelet power spectra under projection and does not carry any restrictions on the embedding and projected dimensions. We show that the wavelet power spectrum of a projected *γ* dimensional measure is $P_{j}=2^{-j\gamma }$, where *j* is the wavelet scale. We contrast the wavelet method with the popular box-counting approach. For projected images, the use of box-counting at fixed thresholds often leads to erroneous results. We apply the method to James Webb Space Telescope (JWST) infrared and Chandra X-ray observations of the supernova remnant Cassiopeia A. We find that the emissions can be represented by projections of mesoscale substructures with fractal dimensions varying from γ=1.69±0.02 for the warm CO layer observed by JWST, up to γ=2.49±0.03 for the hot X-ray emitting gas layer in the supernova remnant.

## Significance

Turbulence is a fundamental process in a wide range of natural and laboratory systems. Experiments and observations often measure projected images of tracers in turbulent flows. Because the underlying structures are fractal, such observations are projections of fractals. This paper presents a general technique for measuring the fractal dimension of structures from their projected images. We apply this method to two-dimensional astronomical images and show that wavelet-based measurements can be used to infer the fractal dimension of a structure in 3D. Applying the technique to JWST infrared and X-ray images of the supernova remnant Cassiopeia A reveals markedly different fractal dimensions for gas at different temperatures, providing a tool for probing the instabilities that generate these complex structures.

Observations of nebulae, molecular clouds, and other astronomical objects reveal intricate structures. In this paper, we discuss how to infer the fractal dimension of a structure in three-dimensional space from projected images. While this work has been motivated by examples from astronomy, our methods are applicable to a broad range of fields.

Many objects show scaling behavior over many orders of magnitude, often generated by hydrodynamical or magneto-hydrodynamical turbulence (1, 2). Astronomical observations show evidence for turbulence spanning up to 12 orders of magnitude (3–6) with the statistical properties of the turbulence depending on Mach number and Alfvenic Mach number (7, 8).

The level sets of tracers in turbulent flows are fractals (9, 10). For astronomical observations, these level sets correspond to the isotherms where emission at a given wavelength peaks. They can arise at boundary layers in a multiphase medium (see e.g., refs. 11 and 12). In burning fronts in combustion engine, the level sets correspond to the gas mixtures are surface of constant gas/oxidant mixture (13).

The dimension of a level set depends on the underlying physics. In studies of combustion, the dimension of the burning front appears to depend on the Damköhler number (14): Flames develop a 7/3 fractal if the chemical reaction time (or equivalently the cooling time) is short compared to the eddy turnover time of the smallest eddies and 8/3 if the eddy turnover time scale is comparable to the chemical reaction time. Simulations (15) suggest that the Kelvin-Helmholtz instability generates a thermal emission layer with dimension 5/2 while simulations of molecular clouds (11) show an index that varies with Alfvenic Mach number. The only information we have is typically a projected image. Thus, if we could infer the dimension of a structure in 3D from its projection, we would have important insights into the physics that determines the observed mesoscale structures.

Astronomers observe the projected images of 3-dimensional object. If the emission at a given frequency, *ν*, is optically thin, then the surface brightness of the image is the projection of the emissivity of the object, $\epsilon_{\nu }$:

[1] $$\begin{array}{c} \Sigma_{\nu }\left(x,y\right)=\int dz\;\epsilon_{\nu }\left(T\left(x,y,z\right)\right). \end{array}$$

Here T(x,y,z) is temperature. We are interested here in the cases like thermal radiative mixing layers (12) where there are sharp temperature changes across a narrow region so that we are observing the projection of a narrow fractal surface rather than extended 3-dimensional structures like HI clouds whose structure can be explored through velocity slicing (16).

Deprojection is generally an ill-posed problem: There are many possible forms of ϵ(x,y,z) consistent with an observed Σ(x,y). Inferring the properties of *ϵ* requires a prior: effectively, a physical model. Our underlying physical model is that we are observing emission from a structure that can be approximated as a *γ*-dimensional surface on its mesoscale, $r_{\min}<r<r_{\max}$. Here, $r_{\min}$ is the thickness of the structure and $r_{\max}$ is the outer scale of the instability that creates the mesoscale phenomenon. Note that Eq. **1** implies that we are interested in measures and their projections, not sets. Indeed, when γ>2, the projection of a set would be typically space-filling, that is, two-dimensional. Observations of a projected set provide no information about the initial structure. Thus, it is important that we are observing a projection (e.g., Eq. **1**). In this paper, we will treat not only the projection from 3 to 2 dimensions but a more general case of the projection from *N* to *D* dimensions.

What is the most effective way of describing the fractal dimension of a measure on the mesoscale? Even with the access to the full structure, not just its projection, this is a very intricate question, and the traditional toolbox is surprisingly misleading. The fundamental problem is that numerical methods developed for “classical” fractals do not accurately capture the mesoscale phenomena in highly disconnected sets under consideration. While box counting methods or equivalently, perimeter-area scalings (17), are widely used to describe sets in fields ranging from engineering (18) to plant science (19) to ophthalmology (20) to astronomy (11, 21) we argue that this approach fails even for the famous problem of determining the properties of a coastline. This method counts the number of boxes, *K*, in $R^{N}$ that contain *μ* as a function of scale, *L* and then defines the Minkowski, or box-counting, dimension as dlogK/dlogL. This approach is problematic (22), particularly if *μ* is not a continuous surface. An important caveat is that in many applications *μ* is fragmented into bubbles. While it may be tempting to introduce a “filling factor” to count the boxes containing *μ*, this is an ill-defined concept: In the limit, the points in *μ* are a set of measure zero in $R^{N}$ and the filling factor, the volume of the box filled by *μ*, is zero. One could say that a bubble is not a point, but when viewed on the mesoscale, a bubble or tidal pool appears as “dust” and box counting breaks down in the presence of “dust.” As an illustration of its failure, consider the rational numbers Q⊂R: Box counting yields a dimension 1 for Q and dimension *N* for $Q^{N}$, that is, it does not distinguish between the set of rational numbers on a line and a line itself. The Hausdorff dimension of the set of real numbers is zero.

If you are a marine biologist who wants to determine the surface area of the tidal zone, you do not want to exclude tidal pools. Similarly, isolated bubbles are not properly counted in analyses of molecular clouds or measurements of the area of a flame front in studies of combustion. In *Measuring Fractal Dimension*, we show that wavelets are a sparse and effective description of a measure that provides a more robust approach than box counting on sets.

An even bigger problem, central to the present paper, is that in astronomy one often only has access to projected images. If one is projecting to $R^{D}$ a set of dimension larger than 2, the result is typically two-dimensional, space-filling, and box counting fails for much more trivial reasons than above: It blindly yields projected dimension 2. The Fourier methods, if attempted, would be contaminated by aliasing. Moreover, we only observe one projection at a time, as opposed to the view from all angles. Thus, the methods of reconstructing the data from projections at all angles used, e.g., in CT, do not apply. This problem is the focus of *Measuring Fractal Dimension from Projected Images*, where the main contributions of the paper are described. We develop a method to infer fractal dimension of a mesoscale structure in $R^{N}$ from observations of projected images in $R^{D}$, under appropriate physical assumptions.

In *Application: Cas A*, we apply this method to an image of the emission from warm CO derived from JWST image of Cas A at F356W and F444W (23, 24) and to X-ray images of Cas A (25).

## Measuring Fractal Dimension

This section focuses on methods for describing the dimension of a measure with either wavelets or Fourier methods. We refer the reader to classical texts, e.g., ref. 22, for rigorous definitions and analysis of some of the mathematical concepts used below.

As elucidated above, Minkowski (box-counting) dimension is not the most effective description of the geometry of disconnected dimensions so we focus on Hausdorff dimension as a tool to describe these structures. We consider a set in $R^{N}$ with a fractional dimension. We will refer to it as a fractal, although we do not assume self-similarity, simply that it exhibits the same dimension across a range of scales. The Hausdorff dimension, *γ*, of a set, is the supremum of *s* so that the Riesz potential,

[2] $$\begin{array}{c} I_{s}\left(\mu \right)=\int \frac{d\mu_{x}d\mu_{y}}{{\left|x-y\right|}^{s}}, \end{array}$$

is finite for some nontrivial and finite Borel measure *μ* on the set.

Wavelets provide an effective language for describing the dimension of a measure. We pick a wavelet basis and let $\phi_{\mathrm{jm}}=2^{jN/2}\phi \left(\left(x-x_{\mathrm{jm}}\right)/2^{-j}\right)$ denote the wavelets concentrated on a cube centered at $x_{\mathrm{jm}}\in 2^{-j}Z^{N}$ of the side length $2^{-j}$. We make sure that wavelets are $L^{2}$ normalized, that is, $\int_{R^{N}}{\left|\phi_{\mathrm{jm}}\right|}^{2}=1$, and denote the amplitude of a wavelet coefficient at scale $2^{-j}$ centered at position $x_{\mathrm{jm}}$ by

[3] $$\begin{array}{c} a_{\mathrm{jm}}=\int \phi_{\mathrm{jm}}d\mu . \end{array}$$

Because fractal dimensional surfaces are sparse and typically not space-filling, wavelets are a sparse representation. In this language, most of the wavelet coefficients (concentrated on boxes that do not intersect the support of *μ*) are close to zero. When, on the other hand, the box corresponding to $\phi_{\mathrm{jm}}$ has an ample intersection with suppμ, generally the amplitude of the corresponding coefficient $a_{\mathrm{jm}}$ scales as $2^{-j\gamma }2^{jN/2}$ (see, e.g., ref. 26, for an analogous computation). Given that *μ* is *γ*-dimensional in $R^{N}$, the proportion of boxes with a nontrivial contribution at every scale is $2^{j(\gamma -N)}.$ Working at mesoscales $r_{\min}<r<r_{\max}$ corresponds to considering wavelets with $-j_{\max}<j<-j_{\min}$. On these scales, the wavelet power spectrum has a power-law behavior:

[4] $$\begin{array}{c} P_{j}=\frac{1}{2^{\mathrm{jN}}}\sum_{m}{\left|a_{\mathrm{jm}}\right|}^{2}\propto 2^{j(\gamma -N)}2^{j(N-2\gamma )}\propto 2^{-j\gamma }. \end{array}$$

Alternatively, we can describe the measure in a Fourier basis:

[5] $$\begin{array}{c} \hat{\mu }\left(k\right)=\int \exp\left(ikx\right)d\mu \left(x\right),\;k\in R^{N}. \end{array}$$

Now, the Riesz potential becomes

$$I_{s}(\mu )=\int |k{|}^{s-N}|\hat{\mu }(k){|}^{2}dk.$$

Hence, bringing us back to the power spectrum, if we assume the power behavior of $|\hat{\mu }{\left(k\right)|}^{2}$, the dimension can be viewed as $\inf\{\tau :|\hat{\mu }\left(k\right){|}^{2}\leq C|k{|}^{-\tau }$}, or more informally, *γ* is the mesoscopic dimension if $|\hat{\mu }{\left(k\right)|}^{2}\approx {\left|k\right|}^{-\gamma }$ for $\frac{1}{r_{\max}}<\left|k\right|<\frac{1}{r_{\min}}$. The description of the measure in Fourier modes is, however, a less sparse representation as the information is spread through Fourier space.

## Measuring Fractal Dimension from Projected Images

How do we deduce the dimension of the original set, *γ*, looking at its projection on $R^{D}$? When γ<D, it almost surely projects into a set of dimension *γ* again, according to Marstand’s 1950 theorem. When γ>D, which is a typical situation discussed in the present paper, the situation is much more delicate. The projection of a set itself would typically be simply *D*-dimensional, space-filling, giving us no information. However, we have richer data. We actually project a *measure* not a set, in the sense that we retain the information of the number of intersections with the set when projecting, but then the challenge is how to take advantage of this information.

Part of the problem is that projection always loses information. Physical systems can have preferred directions determined by either large-scale gravitational fields (e.g., the Earth for oceanographers or atmospheric physicists) or large-scale magnetic fields. If the properties of the set depend on direction, a projection parallel or perpendicular to the symmetry direction yields very different projected images. Luckily, astronomical observations are typically not along a symmetry axis of the system. We can think of this as a form of the Copernican principle that we do not observe the objects in the universe from a special position or orientation.

Projecting the $R^{N}$ wavelet decomposition $\sum_{\mathrm{jm}}a_{\mathrm{jm}}\phi_{\mathrm{jm}}$ to $R^{D}$, we use the property that “horizontally oriented” 3D wavelets $\phi_{\mathrm{jm}}$, $m\in 2^{-j}Z^{N}$, project identically onto 2D wavelets modulo a renormalization: $P\phi_{\mathrm{jm}}=2^{-j\frac{N-D}{2}}\phi_{jm^{♯}}^{♯}$, $m^{♯}\in 2^{-j}Z^{D}$. The “vertically oriented” wavelets project to zero. This would be a problem if horizontal or vertical were a preferred direction, but since we assume that there is none, the information contained in all coefficients $a_{\mathrm{jm}}$ is the same. Hence, we obtain a decomposition of the projected measure,

[6] $$\begin{array}{c} \sum_{jm^{♯}}a_{jm^{♯}}\phi_{jm^{♯}}, \end{array}$$

where $a_{jm^{♯}}$ is the sum of the coefficients $a_{\mathrm{jm}}$ for *m* above $m^{♯}$ times $2^{-j\frac{N-D}{2}}$. For the astronomical images described in the introduction, Eq. **6** is essentially an expansion of Σ(x,y).

In *N* dimensional space on scale *j*, there are $2^{\mathrm{jN}}$ wavelets in a box of size 1. For a *γ* dimensional set, a fraction of roughly $2^{-j(N-\gamma )}$ of the $a_{\mathrm{jm}}$’s are nonzero. These $2^{j\gamma }$ nonzero wavelets project into $2^{\mathrm{jD}}$ wavelets in the projected image. Thus, each $a_{jm^{♯}}$ is a sum of typical *M* amplitudes

[7] $$\begin{array}{c} M\left(j\right)=2^{j(\gamma -D)}={\left(\frac{1}{r}\right)}^{\left(\gamma -D\right)}, \end{array}$$

where *r* is the wavelet scale associated with the *j*-th wavelets. If the coefficients of the modes sum incoherently,^*^ then

[8] $$\begin{array}{cc} a_{jm^{♯}} & \propto M^{1/2}2^{-j\gamma }2^{jN/2}2^{-j(N-D)/2}\\ & \propto 2^{j(\gamma -D)/2}2^{-j\gamma }2^{j\;D/2}\propto 2^{-j\gamma /2}. \end{array}$$

Thus, the dimension of the initial image can be deduced from its *D*-dimensional wavelet decomposition (Eq. **6**) by using the scaling law (Eq. **8**).

**Fig. 1.**
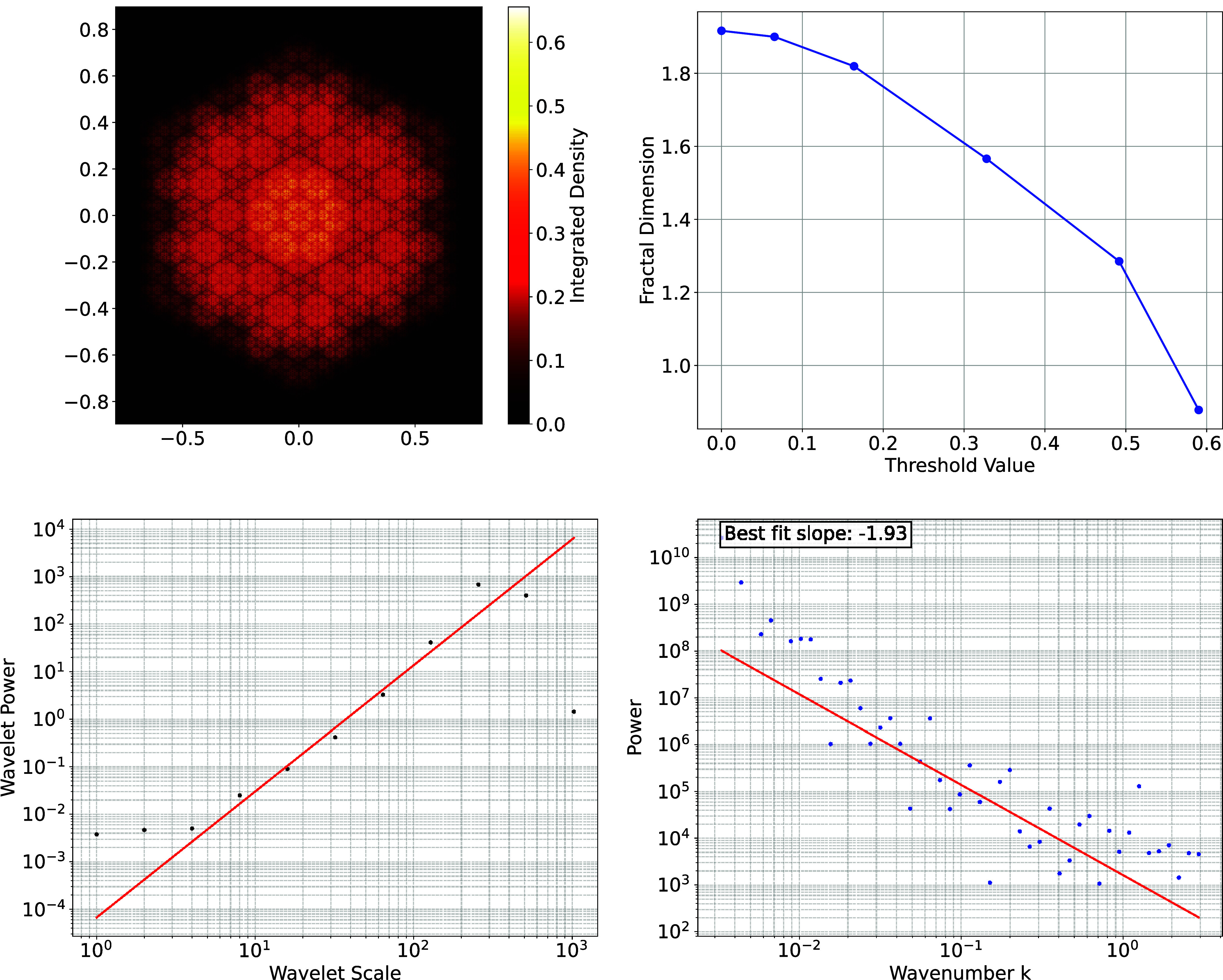
The *Upper Left* panel shows the projected density of a six level Menger sponge, a fractal of dimension γ2.767. The *Upper Right* panel shows the inferred dimension using a threshold method as a function of threshold. The value in this panel should be compared with to γ−1=1.767. The *Lower Left* panel shows the wavelet amplitude versus scale a fit from 4-256 yields a slope of 2.773 (shown) and a fit from 8-128 yields a slop of 2.652. The *Lower Right* panel show the power spectrum of the Fourier transform of the image. The FFT yields 1.912 for the slope corresponding to a dimension of 2.912.

**Fig. 2.**
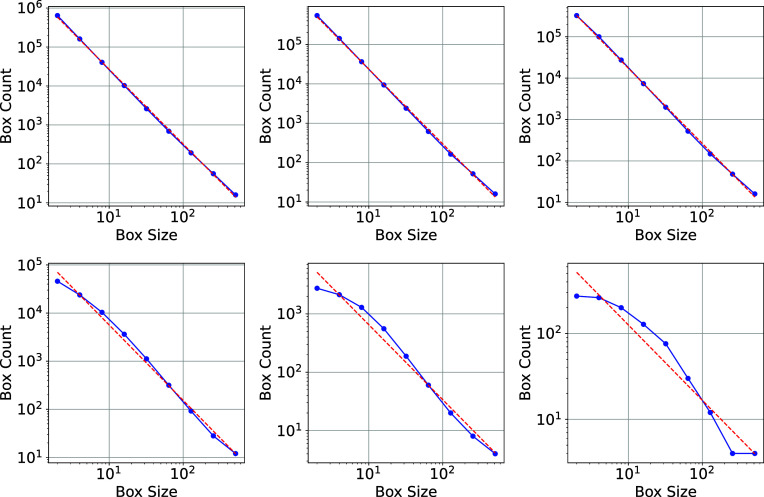
This figure shows the box count versus box size for the projected Menger sponge. For box thresholds set at 0.0, 0.1, 0.2, 0.5, 0.8, and 0.9, the best fit lines (shown as dashed red) have slope 1.902, 1.894, 1.803, 1.521, 1.175, and 0.870. The correct answer should be ∼1.727: While the box counting method can yield convincing power laws, it produces incorrect results when applied to projected density fields.

Fig. 1 shows a projection of a Menger sponge at an angle of 30^°^. The Menger fractal has dimension $\log_{3}\left(20\right)≃2.727$. The figure shows the success of the wavelet method and the limitations of Fourier and box counting methods: Both yield incorrect results. Fig. 2 shows the inferred fractal dimension versus threshold: Box counting can yield a convincing power law for some threshold values but an incorrect result: This may have led to erroneous conclusions in the literature.

Many astronomical objects are projection of complex shapes. At each wavelet position, the path length, $R_{m^{♯}}$, through the three-dimensional structure varies. These leads to a variation in

[9] $$\begin{array}{c} M\left(j,m^{♯}\right)={\left(\frac{R_{m^{♯}}}{r}\right)}^{\gamma -D}, \end{array}$$

However, when we average across the image, this variation does not change the power law scaling.

Averaging over the image, we can define a characteristic thickness, $R_{\mathrm{eff}}$:

[10] $$\begin{array}{cc} {\bar{M}}_{\mathrm{projected}}\left(j\right) & =\frac{1}{2^{\mathrm{jD}}}\sum_{m^{♯}}{\left(\frac{R_{m^{♯}}}{r}\right)}^{\gamma -D}\propto {\left(\frac{R_{\mathrm{eff}}}{r}\right)}^{\left(\gamma -D\right)}\\ & \propto 2^{j(\gamma -D)}. \end{array}$$

Note that $R_{\mathrm{eff}}$ can be defined independently of *j* (only depending on *γ*, *D*, and the shape of the shell). Then again the computation yields the scaling of the wavelet power spectrum:

[11] $$\begin{array}{c} P_{j}^{\mathrm{wavelet}}=\frac{1}{2^{\mathrm{jD}}}\sum_{m^{♯}}a_{jm^{♯}}^{2}\propto 2^{-j\gamma }. \end{array}$$

As long as the path length is large compared to the minimum scale size, the length, $R_{m^{♯}}$ ultimately plays no role in Eqs. **9**–**11**. The wavelets mod out the global structure effortlessly and the dominating shape is not important. This makes it clear why the proposed method is superior to the Fourier decomposition.

Indeed, for a homogeneous uniform slab in Eqs. **6**–**8**, the analysis could be done in Fourier space. Projection in physical space is a slice (restriction) in Fourier space. If we apply the “Copernican principle” and assume that we are not seeing an object from a preferred direction, then the power spectrum of the image in *D* dimensions is just a slice through the *N* dimensional power spectrum. For a projected measure,

[12] $$\begin{array}{c} |{\hat{\mu }}^{♯}\left(k_{D}\right){|}^{2}=Ck_{D}^{-\gamma }, \end{array}$$

where $k_{D}$ is a vector in *D* dimensional space whenever for the original measure $|\hat{\mu }\left(k_{N}\right){|}^{2}=Ck_{N}^{-\gamma }$.

However, an analog of Eqs. **6**–**8** would suffer from the dominating aliasing effects (*Application: Cas A*): One would need to know what is the shape of the initial shell and painfully extract delicate dimension information from an analogue of (Eq. **11**), much less explicit in the Fourier representation. Thus while projection is conceptually simplest in Fourier space, Fourier analysis is poorly suited as even isotropic processes can be inhomogeneous in projection due to these projection effects. As seen in the figure, the Fourier analysis yields the incorrect slope without aliasing corrections. Aliasing corrections can be applied when there is a good model for the three-dimensional object (see e.g., ref. 6). The wavelet-based approach is, however, better suited for more complex structures.

## Application: Cas A

**Fig. 3.**
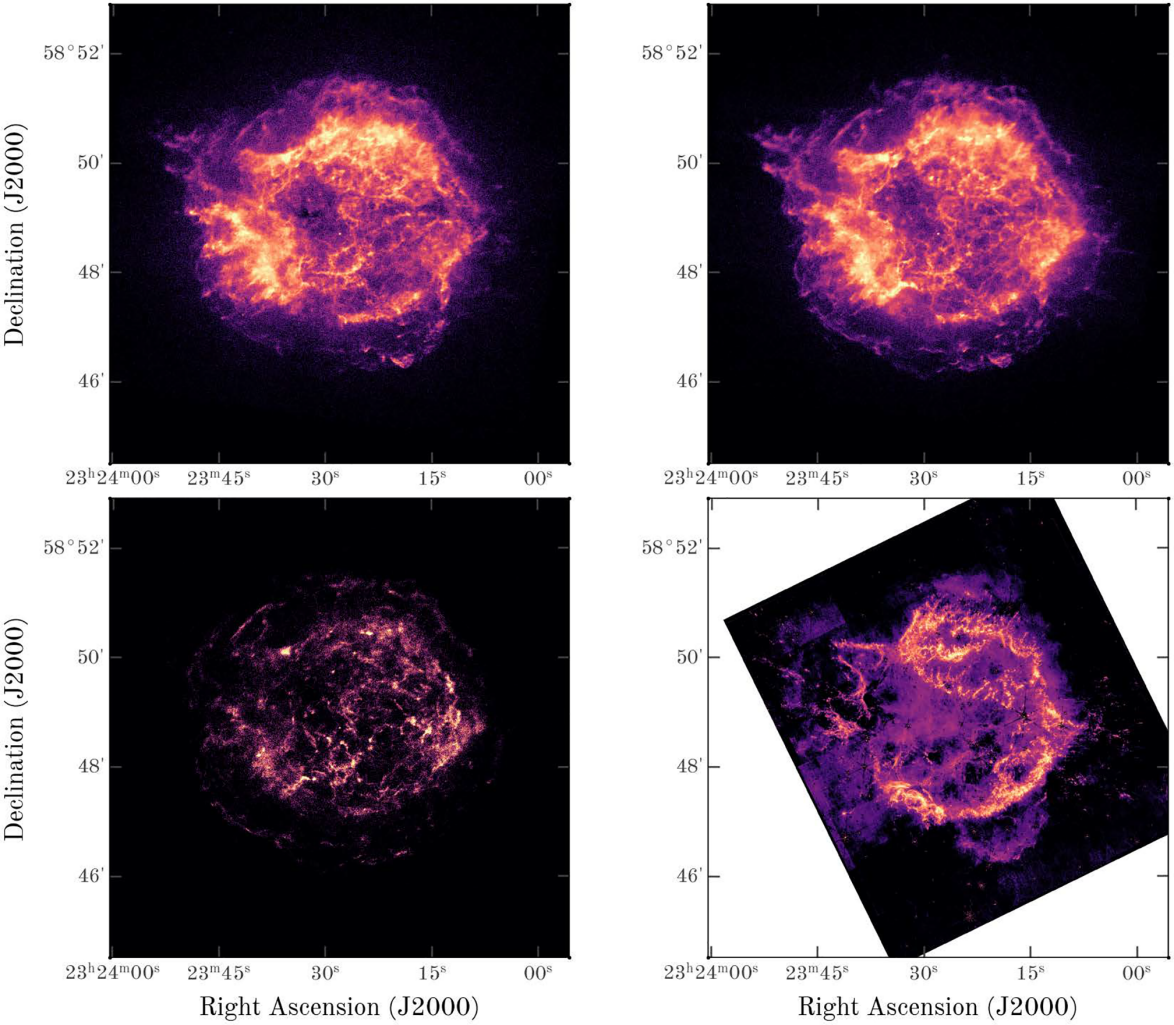
X-ray and Infrared images of Cas A. The first three images are Chandra X-ray images at 0.5 to 1.5 keV, 1.5 to 3.0 keV, and 4.0 to 6.0 keV. The *Lower Right* figure is derived (24) by subtracting the F365W JWST NIRCAM image from F444W JWST NIRCAM image to remove the synchrotron emission. We display the image of the data provided by Tea Temim from the JWST Cas A survey team.

In this section, we apply these methods to a NIRCAM JWST mosaic image of Cas A (23) and to Chandra image at 0.5 to 1.5, 1.5 to 3.0, and 4.0 to 6.0 keV (25). This nearby and young (∼350 y) core-collapse supernova remnant is a particularly interesting case for this study. There are high quality X-ray and near-IR observations that probe the forward and reverse shocks. For over 60 y, physicists have suggested that these shocks are subject to Rayleigh–Taylor instabilities (27). While wavelets have been used to characterize the substructure in X-ray remnants (28), they have not been used to measure fractal dimension. Emission at F444W is dominated by warm CO and synchrotron emission, while emission at F365W is dominated by synchrotron (24). In this section, we analyze an image shown in Fig. 3 that is derived by subtracting the F365W observations from F444W to remove the synchrotron emission (24). We mask negative flux pixels in the synchrotron-subtracted maps provided by Tea Temim from the (23) analysis. These masked pixels are either at the positions of stars or outside the reverse shock. The CO emission in the image is tracing the distribution of warm gas (∼1,000 K) in a reverse shock in the remnant (29). This reverse shock is unstable to Rayleigh–Taylor instabilities and is expected to develop a multiscale fractal-like structure (30).

**Fig. 4.**
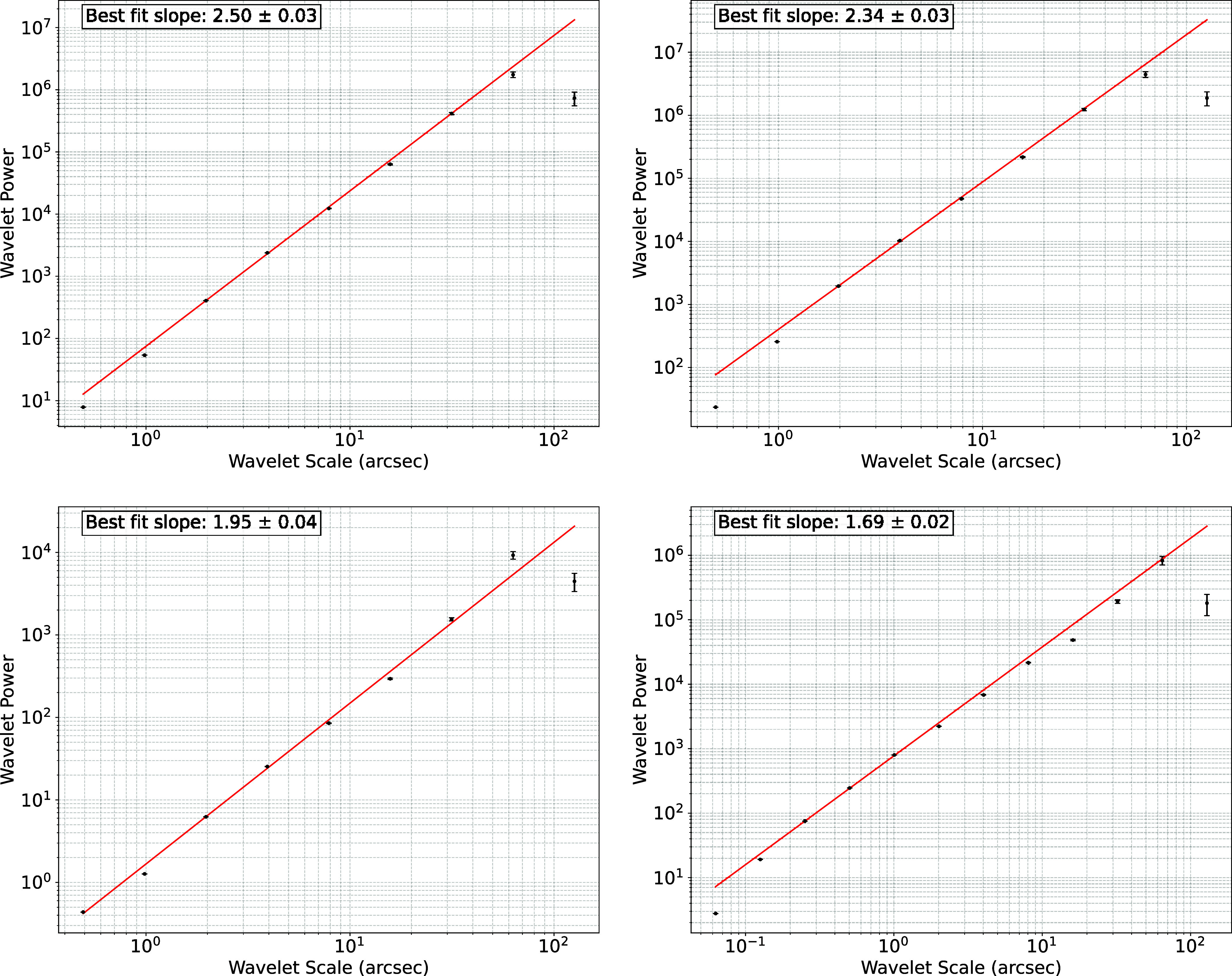
Wavelet Power Spectrum of 2D Image of Cas A for gas at different frequencies. Here, we use Ricker wavelets in the analysis. The *Upper Left* is for 0.5 to 1.5 keV X-ray emission. The *Upper Right* is for 1.5 to 3.0 keV X-ray emission and the *Lower Left* is for 4.0 to 6.0 keV. The *Lower Right* is for the cleaned JWST image. The break from the power law at the largest scales is due to the fractal description of the emitting surface failing at scales comparable to the radius of Cas A. Because of JWST’s better angular resolution, the *Lower Right* spectrum has a larger dynamic range. The error bars are estimated based on the number of modes on a given scale.

Fig. 4 shows the wavelet power spectrum, $P_{j}$, as a function of scale. The wavelet transform of the image shows remarkable power law behavior across a wide range of scales with a spectral index depending on the wavelength. This power law scaling suggests that the description of the image as the projection of fractal emitting layer is consistent with the data. For the CO gas, the slope of the wavelet power spectrum is 1.7. For the X-ray emitting gas, the slope of the power spectrum peaks near 2.5, consistent with simulations of instabilities of a thermal radiative mixing layer (15). Assuming as before that the amplitude of the wavelets are independent and identically distributed, the fractional error on the 2D power spectrum amplitude on scale *l* is $\sqrt{2/N_{\mathrm{modes}}\left(l\right)}$. For a fractal of dimension *γ*, there are ${\left(L_{\max}/l\right)}^{\gamma }$ modes in the object, where $L_{\max}$ is the scale of the system. In fitting the slope, we inverse-variance weight the data. In the fit, we exclude the smallest scale (because of beam smearing) and the largest scale (because it likely is outside the mesoscale range.

It will be interesting to compare numerical studies of the development of instabilities in young supernova remnants (31) to these measurements from the observations.

## Conclusions

In this paper, we establish a method for analyzing images and determining the dimension of projected mesoscale measure, set up in the framework of the wavelet-based analysis. Traditional box-counting methods fail both in projection but in the ambient *N*-dimensional space for disconnected sets. Here, we demonstrate the technique on simulated data and on observations of Cas A, where we find that dimension of the measure depends on the frequency observed. The observations at different frequencies are tracing gas at different temperatures.

We believe this approach is quite general and can be used not only to extract additional information about the underlying physics for a wide range of astrophysical objects, but more generally to analyze data in projection across a variety of fields. For example, it would be interesting to apply these methods to measurements of the intrinsic dimensions of surfaces in high dimensional spaces in machine learning (32) and in measurements of the fractal dimension of decision boundaries.

This work suggests several interesting open questions: What is the relationship between the dimension of the tracer level set and the properties of the velocity field near the boundary? Because of cooling at radiative layers and viscous damping of shear fields near Kelvin-Helmholtz instability boundaries, the properties of the velocity field near the emitting layer may differ from isotropic homogeneous turbulence. How do magnetic fields affect the structure of the boundary layer? Will they produce direction-dependent fractal dimensions? Here we focused on fields projected from 3 to 2 dimensions. Can these techniques be applied to velocity slices in 21-centimeter data (see e.g., ref. 16)?

## Data Availability

All data and codes used in this paper are available on GitHub at DavidSpergel/PNAS2025 (33).

## Footnotes

^*^We thank Guy David for suggesting this $M^{1/2}$ scaling.
